# Development of Conductive Antibacterial Coatings on Cotton Fabrics via Polyphenol-Mediated Silver Mirror Reaction

**DOI:** 10.3390/polym16233244

**Published:** 2024-11-22

**Authors:** Yixiao Wu, Chenlin Fu, Jiaxin Xing, Lin Yang, Chong Zhao, Kun Yan

**Affiliations:** 1 School of Chemical and Environmental Engineering, Wuhan Polytechnic University, Wuhan 430023, China; 2 Key Laboratory of Textile Fiber & Product, Ministry of Education, Wuhan Textile University, Wuhan 430200, China

**Keywords:** polyphenol, silver mirror reaction, cotton fabric, conductivity, antibacterial

## Abstract

Herein, this study reports the development of a multifunctional conductive antibacterial cotton fabric through the utilization of the natural polyphenol-mediated silver mirror reaction. The experimental results demonstrate that polyphenols can effectively facilitate the deposition of silver nanoparticles (AgNPs), resulting in a uniform and durable hybrid nanocoating on the cotton fabric. The effects of polyphenol’s molecular weights on the coating structures and stabilities have been revealed via two distinct approaches: washing resistance and electrochemical testing systems. It has been concluded that lower-molecular-weight phenols induce a compact and dense coating structure, whereas polyphenols such as tannic acid exhibit relatively high stability, achieving an excellent conductivity of 0.2 S/cm and a good washing resistance of 67% over five cycles. The underlying mechanism has been further confirmed by the cyclic voltammetry measurements, suggesting that polyphenols play a significant role in stabilizing AgNPs and preventing their dissolution. Furthermore, the Ag-doped polyphenol-coated fabrics exhibit notable antibacterial properties. By coupling natural polyphenols with typical silver mirror reactions, this study not only offers a sustainable alternative to synthetic chemicals but also presents a promising method to endow cotton textiles with the dual properties of conductivity and antibacterial activity.

## 1. Introduction

Conductive antibacterial textiles (CATs) are crucial for modern applications in smart flexible electronics like healthcare analysis, sportswear monitoring, and remote sensing systems [1,2]. However, developing an effective approach to constructing high-performance textiles with excellent durability and chemical stability still remains a significant challenge [3]. Traditional textiles, which often have limited functionality, lack essential features such as electrical conductivity and antibacterial properties. Commonly, conductive textiles can be easily produced by coating fabrics with various conductive materials, including conductive polymers (e.g., polypyrrole [4], polyaniline [5]), carbon-based nanocomponents (e.g., carbon nanotubes [6], graphene oxide [7], MOFs/COFs [8,9,10], and MXenes [11]), and nanometals [12]. Of these, silver nanoparticles (AgNPs) are particularly noteworthy due to their excellent electrical conductivity and antibacterial properties, making them ideal for constructing multifunctional CATs [13]. Recent reports have demonstrated that the classical silver mirror reaction (SMR) method, traditionally used to create metallic films and coatings, can be adapted for depositing silver nanoparticles onto textile surfaces [14,15,16]. As compared to conventional chemical reduction approach, the SMR offers a faster and more uniform deposition of silver, enhancing its effectiveness for precise applications [17]. However, this method often results in coatings that lack durability and tend to peel off, leading to diminished performance. Thus, there is great interest in developing an effective approach to enhancing the chemical stability of AgNP-doped conductive fabrics.

Natural polyphenols, which are abundant in plants and known for their rich antioxidant properties, have recently gained attention for their potential applications in textile functionalization [18]. Their capacity to act as reducing agents enables the straightforward synthesis of metallic nanoparticles, with silver being an ideal example due to its well-established antimicrobial characteristics [19]. Beyond their role as antioxidants, polyphenols effectively function as reducing agents in chemical reactions, facilitating the formation of metallic nanoparticles, particularly silver [20,21,22]. Additionally, natural polyphenols exhibit remarkable interface adhesion performance due to their ability to form strong interactions with various substrates. The adhesion is primarily facilitated by the functional groups present in polyphenols, such as phenolic hydroxyl (-OH), carboxyl (-COOH), and aromatic rings, which can form hydrogen bonds, ionic interactions, and covalent bonds with the substrate surfaces [23,24,25]. This multifaceted adhesion mechanism ensures that polyphenol-based coatings are robust and highly durable on various hard and/or soft materials. Previous studies have mainly focused on the potential of polyphenols in facilitating metal deposition due to their excellent binding and reducing capabilities [26]. However, little work has been carried out using polymer-mediated silver mirror reactions (SMRs) and optimizing the molecular weight of polyphenols to tailor their deposition properties.

Herein, this research presents a simple and sustainable approach for the green synthesis of CATs by coupling various polyphenols with the silver mirror reaction to mediate the deposition of silver nanoparticles (AgNPs) and tailor their coating properties. The strong interfacial adhesion and binding performance of polyphenols is particularly advantageous in the mediation of nanometal deposition, allowing them to efficiently stabilize and reduce silver ions (Ag^+^) to form AgNPs. Additionally, the use of the silver mirror reaction promises a uniform distribution of AgNPs on the textile surface, ensuring that the antimicrobial properties of silver are effectively utilized. Taking into consideration the development of multifunctional textiles, this work addresses pressing challenges in the fields of flexible electronic devices and smart textile-based sensing.

## 2. Materials and Methods

Materials: A commercial cotton fabric (~138 g/m^2^) was used as the soft substrate for coating treatments. Chemicals: tannic acid (TA, 1701 g/mol), gallic acid (GA, 170 g/mol), curcumin (Cur, 368 g/mol), and dopamine hydrochloride were purchased from Sigma-Aldrich (St. Louis, MI, USA). Sodium hydroxide (NaOH, 96%), ammonia (NH_3_·H_2_O, 25%), silver nitrate (AgNO_3_, 99%), trimethylolaminomethane (Tris), hydrochloric acid (HCl, 37%), and hydrogen peroxide (H_2_O_2_, 30%) were purchased from Sinopharm Group Chemical Reagents Co., Ltd. (Shanghai, China). The Pt wire counter electrodes and Ag/AgCl (3M NaCl) reference electrode were purchased from CH Instruments (Austin, TX, USA). All chemicals were not further purified, and deionized water was used throughout the experiments.

Preparation of the polyphenol-coated cotton fabrics: Initially, the pre-treatment of cotton fabric was conducted by placing a certain amount of cotton fabric into a 1 mol/L NaOH solution and magnetically stirring at about 50 °C for 3 h. Next, the fabrics were rinsed with deionized water until neutral, and then dried at room temperature for later use. Next, a certain amount of the polyphenols (e.g., tannic acid, gallic acid, and curcumin) was weighed and dissolved in a Tris-HCl buffer solution (pH~8.5) to achieve a final concentration of 2 g/L. Specifically, the polyphenol coating was prepared by immersing the pre-treated cotton fabrics in the above polyphenol solutions and reacting in a 40 °C water bath for 12 h. Finally, the fabrics were removed from the solutions and then vacuum-dried at room temperature for 2 h. For clarity, the cotton fabrics coated with tannic acid (TA), gallic acid (GA), and curcumin (Cur) were labeled as n-TA, n-GA, and n-Cur, respectively. Bare cotton fabric with no modification was provided as the control.

Polyphenol-mediated silver mirror reaction (SMR): The ammonia silver solution was first prepared via a previously reported method [17]. Briefly, ammonia was added dropwise to a mixture of silver nitrate (1 M) and NaOH (1 M) until the precipitate completely disappeared. The integration of nano-silvers was carried out by immersing the as-prepared polyphenol-modified cotton fabrics in the above ammonia silver solutions for 8 h and then reacting with a glucose solution (0.1%) for 1 h. After the reaction completed, the fabrics were thoroughly rinsed with deionized water and then dried in a warmed environment at about 35~40 °C for 4 h. For clarity, the Ag-doped cotton fabrics coated with tannic acid (TA), gallic acid (GA), and curcumin (Cur) were labeled as n-TA@Ag, n-GA@Ag, and n-Cur@Ag, respectively. The Ag-modified bare cotton fabric was labeled as n-Ag.

Conductivity and washing resistance: The surface conductivities of Ag-doped fabrics were measured by using an EMAX3 multimeter. Their stabilities were tested via a repeated washing method. Specifically, each process was carried out by washing 5 times with deionized water at 60 °C (about 1 min, stirring speed: 1500 r/min). The washing resistance was calculated from the surface conductivities of the fabrics before and after being subjected to 5 rounds of washing.

Antibacterial performance: The antibacterial activities of the fabrics were determined using a typical disc-diffusion technique [27]. Briefly, two commonly used bacterial strains of *S. aureus* and *E. coli* (10^8^~10^9^ cfu·mL^−1^) were chosen and inoculated separately in Luria–Bertani agar-filled glass Petri dishes (diameter 90 mm). The as-prepared fabrics with varied compositions were promptly placed on the agar surface, and a bare sample fabricated without AgNPs was provided as a control. The inhibition zones were determined after incubation at 37 °C for 24 h and the zone diameter of each sample was calculated from the obtained images by using an image analysis software.

Characterization: The surface morphology and chemical composition of the prepared films were observed by a scanning electron microscope (SEM, Hitachi S-4800, Tokyo, Japan). The crystal phase was studied by a Bruker D8 advanced X-ray diffractometer (2θ = 20~80°) equipped with a Cu Ka radiation source (λ = 1.5406). Using FLIR E6XT 2.1L infrared thermal imager to take temperature records and thermal imager photos. The thermal images of the fabrics were collected by using an TIS75 infrared thermal imager. The water contact angle measurements were conducted by using the sessile drop approach on a Contact Angle System (Dataphysics, Germany KRUSS Co., Hamburg, Germany). The mechanical properties of the fabrics (2 mm × 1 cm × 3 cm) were measured by using a universal testing machine (CMT6350, Shenzhen SANS, Shenzhen, China) at a rate of 0.5 cm/min. Electrochemical measurements such as cyclic voltammetry (CV) were performed using a CHI620E electrochemical analyzer (CH Instruments, Austin, TX, USA) with Ag/AgCl (3M NaCl) as a reference electrode, conductive fabrics as a working electrode, and a Pt wire as a counter electrode. The release of silver ions was evaluated by immersing AgNP-decorated fabrics in water solutions on an inductive coupled plasma–atomic spectrometer (ICP5800, Varian Liberty II AX Sequential, Varian Inc., Palo Alto, CA, USA). The specific experimental conditions are provided in the text and figure captions.

## 3. Results and Discussion

### 3.1. The Natural Polyphenol-Mediated Silver Mirror Reaction Was Performed on Cotton Fabric

**Figure 1:**
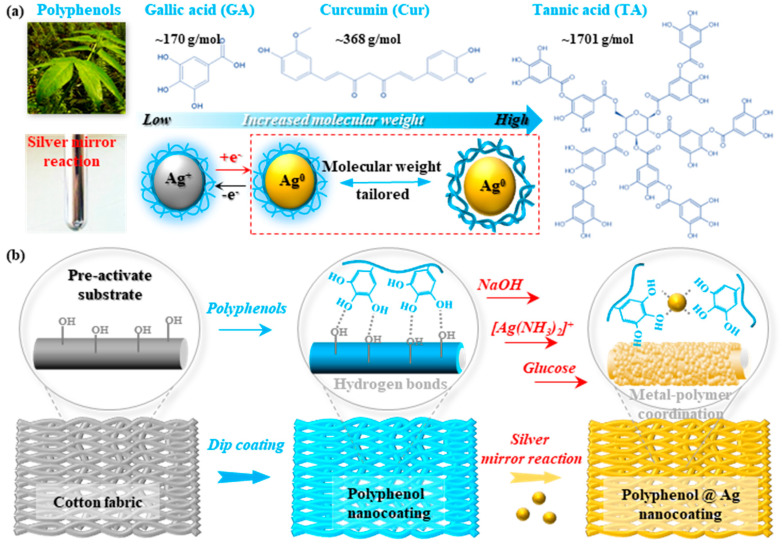
(**a**) The chemical structures of natural polyphenols and the effects of their molecular weights on the structures of polyphenol@Ag nanocoatings. (**b**) The schematic illustrates the preparation processes and polyphenol-mediated self-assembly of AgNPs on the cotton fabric.

The widespread attention to flexible wearable textiles has placed higher demands on the materials themselves, requiring superior conductivity, stability, antibacterial properties, skin-friendliness, energy efficiency, and environmental sustainability. These materials must also be capable of seamlessly integrating with the human body, ensuring both comfort and functionality [2]. Therefore, as shown in Figure 1a, an innovative approach was developed to enhance the properties of cotton fabrics by using various natural polyphenols with different molecular weights (i.e., ranging from 170 g/mol to 1701 g/mol) to mediate the deposition of silver nanoparticles (AgNPs). Traditionally, textile enhancement involves direct silver plating, which often results in limited control over the deposition process and uneven coatings [28]. Recently, natural polyphenols have received more attention in terms of mediating the deposition of nanometals due to the abundant catechol groups on their chains’ backbones. The polymer matrix could provide abundant active sites for metal ion immobilization and then catalyze the reduction to the corresponding nanometals (e.g., Ag^+^ to Ag^0^) via its inherent redox activities and/or other chemical reductants (e.g., NaBH_4_) [29]. To the best of our knowledge, therefore, this is the first attempt to combine polyphenols and classical silver mirror reactions, particularly by controlling their molecular weight, to achieve a more uniform and stable deposition of silver nanoparticles (AgNPs). The underlying mechanisms and the polyphenol-mediated silver mirror reaction are displayed in Figure S1, and the silver ions are reduced to AgNPs by glucose in ammonia solutions. Initially, as shown in Figure 1b, the pre-treatment of cotton fabric was conducted by placing a certain amount of cotton fabric into a 1 mol/L NaOH solution and magnetically stirring at about 50 °C for 3 h. Specifically, the NaOH solution can react with cotton fibers through saponification, thereby activating the functional hydroxyl groups and removing impurity adhesion on the fabric surface to improve the polyphenol coating efficiency. Additionally, the employment of the silver mirror reaction (SMR) allows for finer control over the size and distribution of silver particles, significantly improving the electrical conductivity of the coated fabric. Therefore, in this study, coupling different natural polyphenols with the typical mirror reactions not only enhances the conductivity of cotton fabrics, making them suitable for various electronic applications, but also improves the stability and durability of the coating under mechanical stress and environmental exposure.

### 3.2. Surface Morphologies and Characterizations

**Figure 2:**
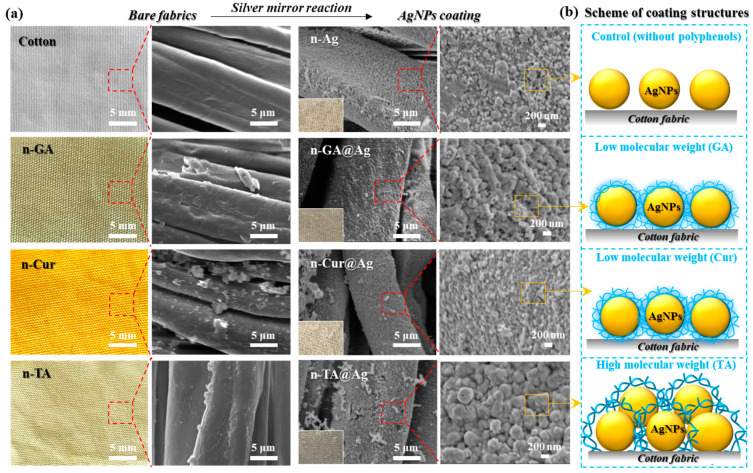
(**a**) Optical and SEM images of the cotton fabrics before and after modification with different natural polyphenols and AgNPs. (**b**) The schematic illustrates the possible structures of nanocoatings prepared from natural polyphenols with different molecular weights.

To test the above hypothesis, as shown in Figure 2, a series of optical images and corresponding microstructural observations were collected to confirm the successful preparation of natural polyphenol coatings such as tannic acid (TA), gallic acid (GA), and curcumin (Cur), followed by the introduction of AgNPs through a typical SMR approach. It can be seen that the uncoated cotton fabric presents a smooth and uniform fiber surface. As expected, the application of these polyphenols induced distinct changes in the fabric’s appearance and microstructure, each imparting a unique coloration and texture. The SEM results indicate the fiber surfaces became rougher, facilitating the subsequent silver deposition within such an additional coating layer. After the reaction completed, a uniform AgNP cake layer could be found on the polyphenol-modified cotton fabrics with both a typical granular and textured surface, signifying the successful formation of silver nanoparticles. These results are in good agreement with the theory that AgNP coatings could be prepared on a certain substrate with a uniform distribution via the silver mirror reaction [14].

The coatings resulting from this process highlight the enhanced adhesion and distribution of silver particles facilitated by polyphenols [30]. The substantial increase in surface area is advantageous for boosting conductivity and antimicrobial properties. Tannic acid, gallic acid, and curcumin serve as critical stabilizing agents, adeptly controlling the nucleation and growth of silver nanoparticles to create a well-dispersed and robust nanocoating [31,32]. Therefore, this approach offers an environmentally friendly and scalable solution for fabric functionalization and represents a significant advancement over conventional methods [33,34]. Moreover, the AgNP cake layer shows a relatively confined and compact coating structure from the modified samples, indicating that the polyphenols could facilitate the deposition of AgNPs. Interestingly, n-GA@Ag and n-Cur@Ag both exhibit a well-confined AgNP coating layer as compared to n-TA@Ag. This result could be possibly attributed to the fact that the lower-molecular-weight phenolics (GA and Cur) can induce the more compact accumulation of AgNPs on the fiber surfaces and the formation of a continuous uniform particle-based conductive network (Figure 2b). The EDS analysis of n-TA@Ag is provided in Figure S2, indicating a strong presence of Ag on the fabric surface. However, the high molecular weight of natural polymers, such as tannic acid (TA), can achieve a more robust AgNP cake layer with good conductivity and enhanced durability. Therefore, this section confirms the great possibility of tailoring the microstructures and properties of nanocoatings on cotton fabrics via the use of different polyphenols and the SMR approach.

**Figure 3:**
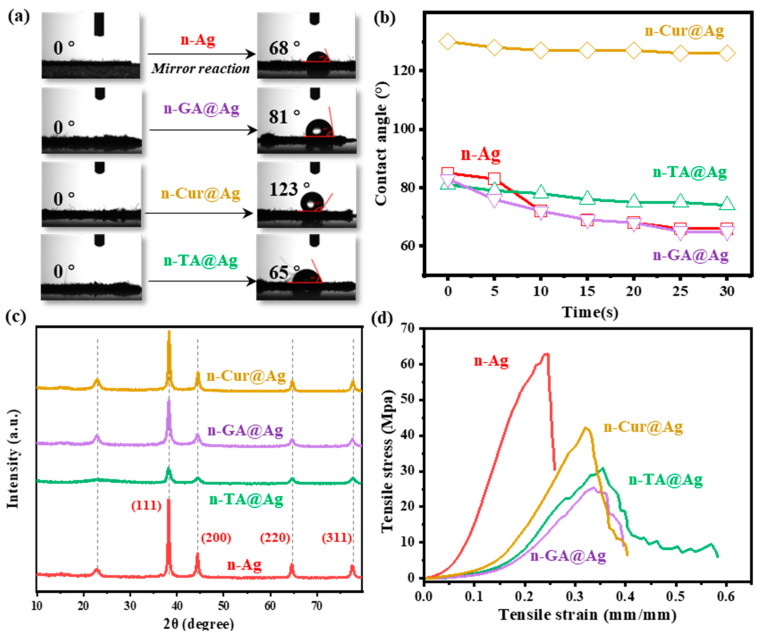
(**a**) Optical images, (**b**) water contact angles, (**c**) crystalline structures (XRD), and (**d**) mechanical properties of the cotton fabrics decorated with different components.

Next, the chemical and mechanical properties of the as-prepared nanocomposites are investigated in Figure 3, providing a comprehensive overview of the enhancements of the functional properties of cotton fabrics treated with polyphenol@Ag nanocoating. The water contact angle measurements displayed in Figure 3a,b exhibit a significant transformation in surface wettability, with the untreated cotton showing high hydrophilicity while the coated fabrics exhibit increased hydrophobicity, particularly with n-Cur@Ag achieving a contact angle of 123°. This improvement is crucial for applications requiring water resistance, such as outdoor textiles or medical fabrics, aligning with the literature that indicates reduced surface energy and enhanced hydrophobicity with the incorporation of hydrophobic agents.

The crystalline structures and mechanical properties further elucidate the impact of the treatments on the cotton fabrics. The X-ray diffractograms are shown in Figure 3c. The characteristic diffraction peaks for Ag-doped fabrics observed at the positions of 37.8°, 44.21°, 64.20°, and 77.50° are attributed to the (211), (200), (220), and (311) planes of the fcc silver, respectively [35,36]. The samples exhibited various peak intensities, indicating that the *AgNPs* were successfully synthesized on the polyphenol-coated cotton fabrics via the silver mirror reactions. Moreover, it can be concluded that the Ag nanoparticles were successfully synthesized and evenly distributed on the cotton fiber surfaces by using different polyphenols as the stabilizers and coating matrixes. Compared with the n-Ag sample, the intensities of the characteristic peaks for Ag-doped polyphenol-modified cotton fabrics progressively decreased with the molecular weights of the natural polyphenols, suggesting that polyphenol nanocoating can tailor Ag mobilization capacities and production efficiency. The mechanical testing results are displayed in Figure 3d. It can be seen that while there are variations in tensile strength and elasticity, the treatments maintain or enhance mechanical integrity without significant degradation.

### 3.3. Investigation of Photothermal Performance

**Figure 4:**
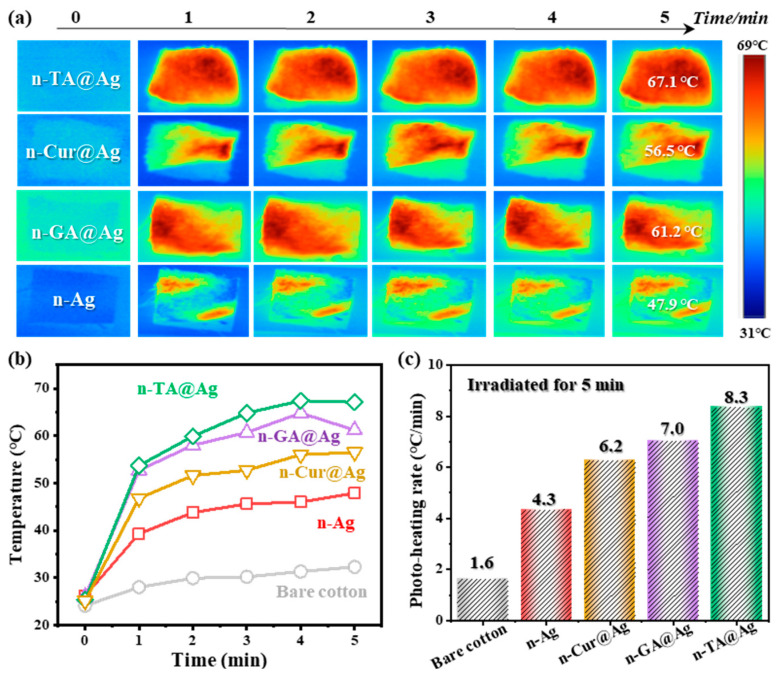
(**a**) Photothermal images of the as-prepared fabrics irradiated with an infrared (IR) lamp for 5 min; the power density is about 0.5 W cm^−2^. (**b**) The surface temperature as a function of irradiation time and the compositions of the fabrics. (**c**) Photo-heating rates of the fabrics with different nanocoating treatments.

To reveal the notable differences in heating efficiency and surface temperature among the treated fabrics, the photothermal images are shown in Figure 4a. It can be seen that all coated fabrics exhibited an increase in temperature under IR irradiation for over 5 min. These results could be attributed to the surface plasmon resonance effects of AgNPs, resulting in an enhanced photothermal conversion capability. In particular, fabrics coated with Ag show higher temperature elevations, which could be further improved by the polyphenols. These results imply that polyphenols facilitate the formation of silver nanoparticles, and result in an improved thermal response to the cotton fabric.

The changes in the surface temperature of fabrics were quantitatively investigated as a function of irradiation time. Figure 4b clearly shows the superior photothermal performance of n-TA@Ag, n-GA@Ag, n-Cur@Ag, n-Ag, and bare cotton, for which maximum temperatures of 67.1 °C, 61.2 °C, 56.5 °C, 47.9 °C, and 32.3 °C were achieved, respectively. This enhancement is likely due to the efficient light absorption and conversion properties of both the AgNPs and the polyphenol matrices, which promote rapid heat generation. Interestingly, n-TA@Ag possesses the maximum conversion efficiency, achieving a photo-heating rate of 8.3 °C/min over 5 min (Figure 4c). This could be explained by the typical supramolecular structure of the TA nanocoating, resulting in a thicker layer matrix with more AgNPs immobilized and enhancing the photothermal efficiency. This behavior is consistent with the hypothesis concluded from Figure 2 that the polyphenols with higher molecular weights would lead to a thicker coating matrix. Overall, this enhanced photothermal conversion efficiency is a feature that can be highly beneficial for winter wear or therapeutic applications.

### 3.4. Assessment of Washing Resistance and Conductivity

**Figure 5:**
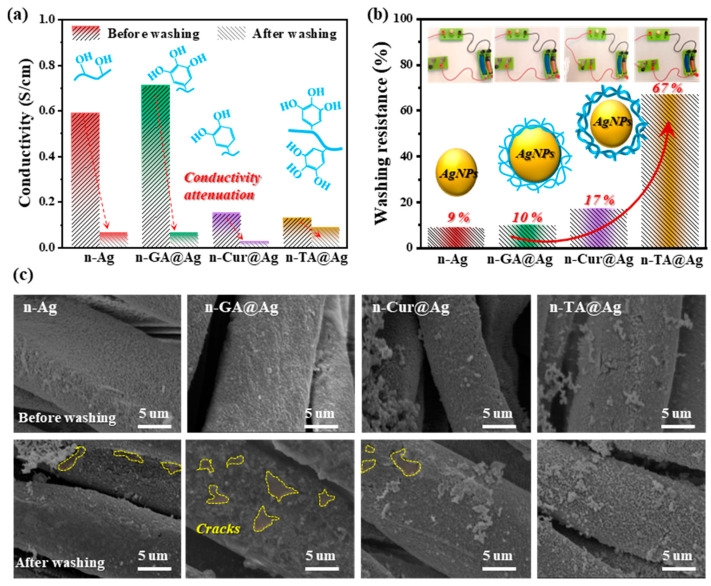
(**a**) Conductivities of the fabrics before and after being washed in water at 60 °C for five cycles (each cycle lasting about 1 min, stirring speed: 1500 r/min). (**b**) Assessment of the stability of the fabric’s conductivity after five rounds of washing; the inserted images indicate the high conductivity that allowed the fabric to light up a LED light. (**c**) Surface morphologies of the fabrics before and after washing treatments.

Durability is critically important in wearable electronic applications, where sustained conductivity through repeated washing is a major hurdle. To reveal the stability for practical applications, the conductivity and washing resistance of the nanocomposite coatings on cotton fabrics were tested. As shown in Figure 5a, all fabrics initially exhibited high conductivities ranging from 0.13 S/cm to 0.7 S/cm due to the incorporation of AgNPs and relatively compact coating structures. These results are in good agreement with those of high-performance AgNP coatings based on the advantages of the mirror reaction. However, after undergoing multiple wash cycles at 60 °C, the efficacy of these coatings became markedly distinct. n-TA@Ag stood out with superior conductivity retention, emphasizing the robust adhesion and stabilization effects of this particular polymeric coating. The superior performance of n-TA@Ag can be traced back to tannic acid’s unique binding capabilities and super-molecular structure, which enhance the structural integrity of the conductive pathways. In terms of stability assessments (Figure 5b), n-TA@Ag coatings demonstrate exceptional resilience against multiple washings, maintaining their conductive properties without significant degradation (67%). This outstanding performance is likely rooted in the strong interaction between polyphenols and silver nanoparticles, forming a stable conductive network, a phenomenon corroborated by previous studies on biocompatible binders [37].

The scanning electron microscopy (SEM) images presented in Figure 5c offer a visual comparable observation of the fabrics’ surface morphologies before and after washing cycles. Prior to washing, all samples exhibited uniformly distributed nanoparticles across the fiber surfaces. Conversely, the post-wash SEM images reveal varying levels of nanoparticle detachment and surface degradation. n-Ag without polyphenol treatment exhibits a notable detachment and many surface cracks, indicating weaker adhesion between the nanoparticles and the fiber surface. This contrasts sharply with the n-TA@Ag-treated fabrics, which maintain a more intact surface morphology, reinforcing the earlier findings on their conductivity retention. Morphological resilience is essential for ensuring long-term performance and is consistent with reports that robust surface coatings can effectively mitigate mechanical stress and environmental degradation [38].

**Figure 6:**
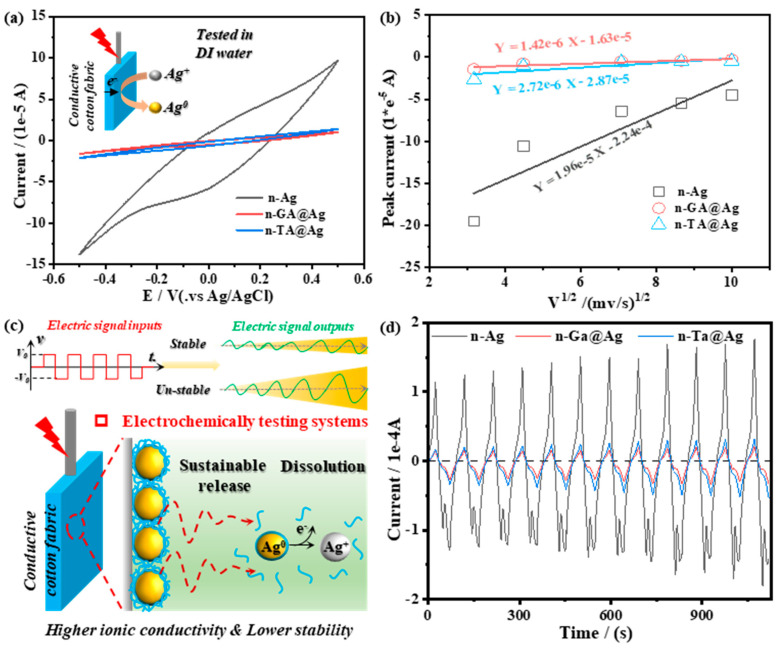
(**a**) The cyclic voltammetry (CV) measurements were conducted using conductive fabrics as the working electrodes in a distilled water (DI) solution (20 mV/s). (**b**) The peak currents as a function of the square root of the scan rate and the corresponding linear fitting results. (**c**) Schematic illustration of the mechanisms of the electrochemical tests used to reveal the relationship between ionic conductivity and coating stability. (**d**) The cyclic voltammetry curves of the conductive fabrics over 12 rounds.

A conventional electrochemical cell was used in the initial electrochemical characterization which used conductive fabrics (2 mm × 1 cm × 3 cm) as the working electrode with a Pt wire counter electrode, and a Ag/AgCl (3 M) reference electrode. To investigate the stability of the conductive fabrics, a specific deionized water solution was initially added to the electrochemical cell and N_2_ gas was purged for 15 min to avoid interference from O_2_ reduction reactions. The results depicted in Figure 6 provide a valuable insight into the conductivity performance and stability of the fabrics through cyclic voltammetry (CV) analysis. Figure 6a showcases the CV measurements of the conductive fabrics serving as working electrodes in a deionized water solution, operated at a scan rate of 20 mV/s. A stable rectangular shape in such CV curves typically indicates good capacitive behavior, which is vital for applications requiring consistent conductivity and energy storage capabilities. The shape and area under these curves can help infer the electrochemical stability of the polyphenol-mediated AgNP coatings. In Figure 6b, the peak currents are plotted against the square root of the scan rates, yielding a linear relationship only for both n-GA@Ag and n-TA@Ag. This correlation suggests that the electrochemical processes occurring within the coatings are diffusion-controlled, and further confirms the great stability of the polyphenol@Ag coatings. According to the Randles–Sevcik equation, a linear relationship of this nature implies that the ion diffusion is consistent and predominantly governs the charge transfer kinetics in these systems [39]. As compared to the non-linear fitting results of the n-Ag control sample, this diffusion-controlled behavior of polyphenol@Ag is indicative of stable and well-adhered coatings where the ion transfer does not face significant resistance, highlighting their robustness.

Figure 6c schematically illustrates the mechanisms underpinning the electrochemical tests. It emphasizes how the interaction between ionic conductivity and coating stability can be assessed through electrochemical inputs and outputs. The n-Ag working electrode was expected to be unstable and allow the dissolution of AgNPs located on the textile surface, leading to a sustainable release of silver ions and improved conductivity for the bulk water solution. These results could be further confirmed by the cumulative release of Ag ions from the polyphenol–Ag-coated fabrics (Figure S3) The stability of the coatings can be inferred from the consistent signal outputs despite external perturbations. Figure 6d shows the CV curves over 12 consecutive cycles, illustrating the conductive fabric’s long-term electrochemical stability. The relative constant peaks and current responses observed from n-GA@Ag and n-TA@Ag imply that the coatings maintain their structural and functional integrity, resisting degradation or significant changes in electrochemical behavior. These results indicate that the Ag-doped polyphenol nanocoating maintain consistent ion exchange and electron transfer processes. Similar findings can be found in other studies, such as those involving graphene or carbon-based conductive materials [40]. The sustained performance suggests that the integration of polyphenols can effectively enhance t particle–fabric interactions, resilience against dissolution, and effective conductive pathways. The use of natural polyphenols likely plays a vital role in forming these stable coatings, offering an environmentally friendly alternative to synthetic methods.

### 3.5. Antibacterial Activities of Polyphenol/Nano-Silver-Coated Decorated Cotton Fabrics

**Figure 7:**
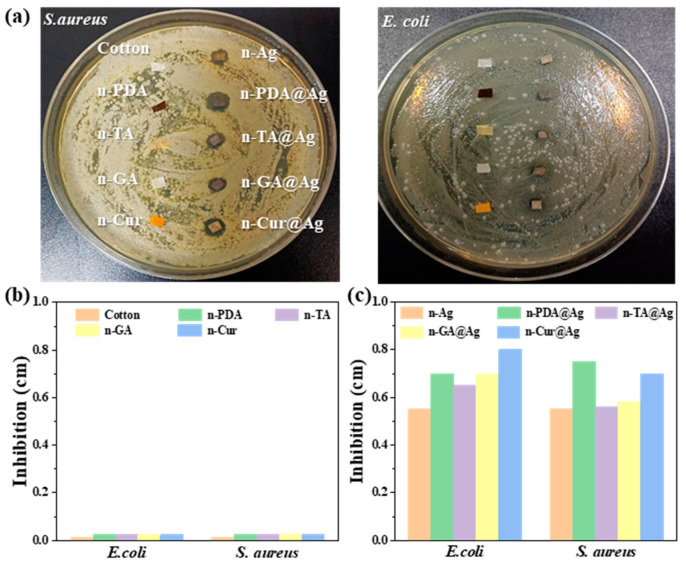
Antibacterial tests of the fabrics against two commonly used bacterial strains: *S. aureus* and *E. coli*. (**a**) Agar plate tests of different fabrics co-cultured with the two bacteria at 37 °C for 24 h. Inhibition zone diameters of the fabrics decorated with (**b**) natural polyphenols and (**c**) natural polyphenol/nanometal composites.

The antibacterial tests depicted in Figure 7 provide substantial insights into the efficacy of fabrics treated with natural polyphenols and polyphenol/nanometal composites against two commonly used bacterial strains. The formation of inhibition zones around the fabric samples serves as a quantitative measure of their antibacterial properties. Untreated cotton, serving as a control, exhibits no inhibition zones, highlighting the necessity of additional treatments to endow fabrics with antimicrobial properties, which are crucial for applications in flexible electronic sensors and wearable textiles. In Figure 7a, fabrics treated with individual natural polyphenols such as polydopamine (PDA), tannic acid (TA), gallic acid (GA), and curcumin (Cur) displayed limited antibacterial activities, as indicated by a small or absent inhibition zone. This observation aligns with previous research suggesting that while polyphenols possess some antibacterial effects, their efficacy is often limited by factors such as poor solubility and weak interaction with bacterial cell walls [41]. Despite these limitations, the use of natural polyphenols remains valuable due to their low toxicity and potential synergistic interactions with nanometals. Significantly, Figure 7a,b demonstrate a substantial enhancement in antibacterial activity when these polyphenols are combined with AgNPs. Fabrics treated with polyphenol/silver composites, such as n-PDA@Ag, n-TA@Ag, n-GA@Ag, and n-Cur@Ag, exhibit much larger inhibition zones compared to those treated with polyphenols alone. The proposed antibacterial mechanisms are provided in Figure S4. Antibacterial reagents such as silver ions and ROS generate the sustainable dissolution of AgNPs and oxidation of catechol groups in polyphenols, which are reported to disrupt bacterial cell membranes and interfere with metabolic processes, resulting in a synergistic antibacterial effect [24]. These findings highlight the significance of advancing multifunctional fabrics, particularly in the domain of wearable electronics, where antimicrobial properties are crucial for user safety and comfort. Future research could focus on optimizing the concentration and dispersion of nanometal particles to maximize conductivity and antibacterial activities while maintaining fabric flexibility, further broadening the scope of antibacterial textiles for various high-demand sectors.

## 4. Conclusions

In summary, a multifunctional conductive antibacterial cotton fabric has been successfully developed through the synergistic use of natural polyphenol coatings and the silver mirror reaction (SMR). This study represents the first application of natural polyphenols in mediating SMR, yielding compact, durable coatings on cotton fabrics with superior conductivity and stability. The SMR process ensures a uniform silver nanoparticle (AgNP) coating on the fabric, while polyphenols enhance interfacial adhesion to the textile substrate. The resulting coatings exhibit remarkable conductivity (0.2 S/cm) and robust antibacterial properties, effectively meeting the dual requirements for advanced textile applications. Furthermore, the impact of phenols with varying molecular weights on coating structure and stability has been elucidated via washing resistance tests. Phenols with lower molecular weights promote a compact, dense coating, whereas high-molecular-weight polyphenols, such as tannic acid, demonstrate higher stability, maintaining a conductivity of 0.13 S/cm and retaining 67% washing resistance after five cycles. The cyclic electrochemical tests confirm that polyphenols stabilize AgNPs and prevent dissolution as compared to the bare Ag-doped cotton sample. Future research may focus on optimizing the coating process and exploring additional functionalities to enhance practical applications. Overall, this work represents significant progress in textile engineering, offering eco-friendly solutions to augment fabric functionality and positioning this technology at the forefront of functional textile development.

## Data Availability

The original contributions presented in the study are included in the article and Supplementary Material, further inquiries can be directed to the corresponding author.

## Supplementary Materials

The following supporting information can be downloaded at: https://www.mdpi.com/article/10.3390/polym16233244/s1, Figure S1: Schematically illustrate the underlying mechanisms and the polyphenol-mediated silver mirror reaction, where silver ions are reduced to AgNPs by glucose in ammonia solutions; Figure S2: EDX measurements of the C/O/Ag element distribution on n-TA@Ag; Figure S3: (a) Calibration curves of silver metal ions show the linearly correlations between the intensities and concentrations. (b) Cumulative Ag ions released from the fabrics of n-Ag, n-GA@Ag and n-TA@Ag; Figure S4: Proposed synergistic antibacterial properties based on sustainable dissolution of AgNPs and release of reactive oxygen species (ROS) generated from the redox reactions of catechol groups in polyphenols.
