# Supplementary material for: Development of Conductive Antibacterial Coatings on Cotton Fabrics via Polyphenol-Mediated Silver Mirror Reaction
Source: Polymers (Basel). 2024 Nov 22;16(23):3244. doi: 10.3390/polym16233244 (PMC11644306; doi:10.3390/polym16233244)
Supplement: Supplementary file 1 [file polymers-16-03244-s001.zip › polymers-3298157-supplementary.pdf]

Supporting Information

Yixiao Wu [1], Chenlin Fu [2], Jiaxin Xin [2], Lin Yang [1], Chong Zhao [1] and Kun Yan [2,*]

[1] School of Chemical and Environmental Engineering, Wuhan Polytechnic University, Wuhan 430023, China
[2] Key Laboratory of Textile Fiber & Product, Ministry of Education, Wuhan Textile University, Wuhan 430200, China
* Correspondence: kyan@wtu.edu.cn

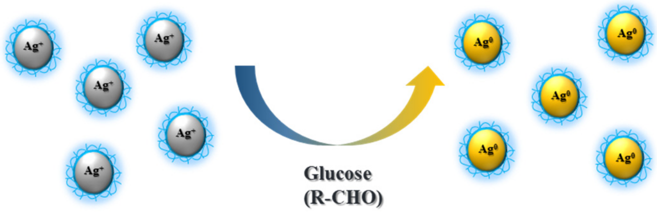

Euq 1： $Ag^{+}+NH_3 \rightarrow Ag(NH_3)_2$

Euq 2： $R\text{-}CHO+2Ag(NH_3)_2{+}+3OH^{-} \rightarrow R\text{-}COO^{-}+2Ag+4NH_3+2H_2O$

Figure S1. Schematically illustrate the underlying mechanisms and the polyphenol-mediated silver mirror reaction, where silver ions are reduced to AgNPs by glucose in ammonia solutions

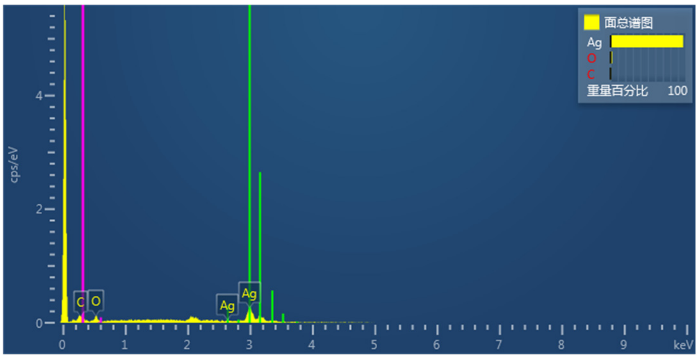

Figure S2. EDX measurements of the C/O/Ag element distribution on n-TA@Ag.

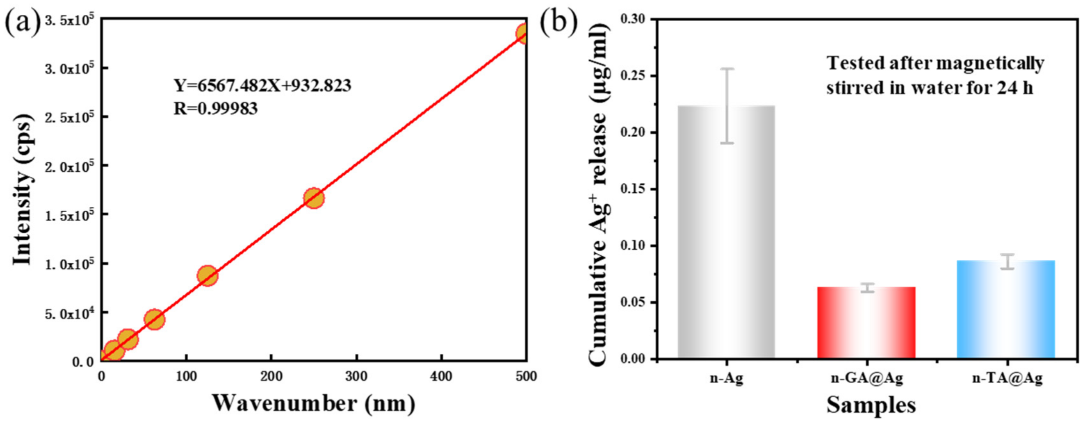

Figure S3. (a) Calibration curves of silver metal ions show the linearly correlations between the intensities and concentrations. (b) Cumulative Ag ions released from the fabrics of n-Ag, n-GA@Ag and n-TA@Ag.

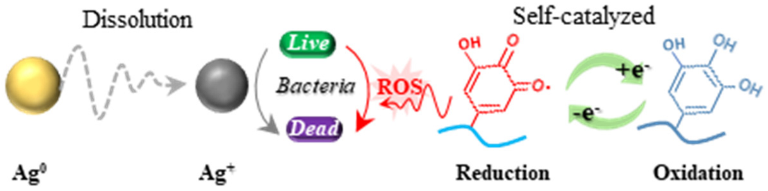

Figure S4. Proposed synergistic antibacterial properties based on sustainable dissolution of AgNPs and release of reactive oxygen species (ROS) generated from the redox reactions of catechol groups in polyphenols.
